# Posterior C1-2 Fusion Using Large-Diameter C2 Pars and Intralaminar Screws in the Setting of a High-Riding Vertebral Artery: A Case Report

**DOI:** 10.7759/cureus.104688

**Published:** 2026-03-04

**Authors:** Yuhi Yoshikawa, Takahiro Mori, Satoshi Maki, Takashi Hozumi, Seiji Ohtori

**Affiliations:** 1 Department of Orthopaedic Surgery, Chiba University, Graduate School of Medicine, Chiba, JPN

**Keywords:** atlantoaxial subluxation, c2 pars screw, down syndrome, high‐riding vertebral artery, intralaminar screw

## Abstract

A high-riding vertebral artery (HRVA) narrows the C2 pedicle corridor and increases the risk of vertebral artery injury during C2 pedicle or transarticular screw placement. Selecting an anchor that preserves safety without compromising stability remains challenging in such cases.

A 32-year-old man with Down syndrome presented with neck and left-sided limb pain, left-hand clumsiness, and gait disturbance. Imaging showed atlantoaxial subluxation (AAS) with left HRVA. We performed posterior C1-2 fusion using a large-diameter C2 pars screw together with a C2 intralaminar screw on the HRVA side, plus a C1 lateral mass screw and a contralateral C2 pedicle screw. Postoperatively, symptoms improved, and no implant-related complications were observed at one-year follow-up.

In AAS complicated by HRVA, a large-diameter C2 pars plus intralaminar screw construct can secure a safe trajectory while achieving satisfactory initial stability and clinical improvement. This hybrid strategy may serve as a practical alternative when a C2 pedicle trajectory is unsafe.

## Introduction

Atlantoaxial subluxation (AAS) can progress to myelopathy and pain when canal compromise persists. C2 pedicle or transarticular screws provide strong fixation but carry a risk of vertebral artery injury, particularly when a high-riding vertebral artery (HRVA) narrows the osseous corridor [1-3]. Alternative anchors, including C2 laminar or pars screws, are viable options with comparable construct stability in selected contexts [4,5]. Biomechanical and finite-element studies suggest that screw diameter and cortical purchase are major determinants of pullout strength and construct robustness [6,7].

Despite these insights, anchor selection in HRVA-complicated AAS remains debated. Pars screws reduce vascular risk by shortening the trajectory, yet can be mechanically inferior to pedicle screws unless purchase is optimized [8]. Hybrid strategies that augment a pars-based construct with a C2 intralaminar screw (ILS) may offset directional weaknesses and distribute load across multiple cortices; however, radiographically detailed real-world examples remain limited [9].

Here, we present a young adult with Down syndrome and HRVA-complicated AAS treated by a posterior C1-2 fusion using a large-diameter C2 pars screw combined with a C2 ILS. We describe the measurements guiding anchor choice, emphasize cortical purchase, and show postoperative improvement in C1-2 alignment and symptoms, offering practical guidance when a C2 pedicle trajectory is unsafe.

## Case presentation

A 32-year-old man with Down syndrome developed neck and left-sided limb pain accompanied by left-hand clumsiness and gait disturbance several months before his first visit to our hospital. He was initially evaluated at an outside hospital and was referred to our department because his symptoms persisted. There was no history of cervical trauma or rheumatologic disease, and family and social histories were noncontributory. Because he had difficulty following commands, manual muscle testing could not be reliably performed, but no obvious limb weakness was documented. The left lower extremity showed clearly hyperactive deep-tendon reflexes with ankle clonus, and other elements of the neurological examination could not be fully quantified for the same reason. Objective scoring for myelopathy, such as the Japanese Orthopaedic Association (JOA) score or Nurick grade, could not be formally assessed due to the patient's cognitive impairment associated with Down syndrome.

**Figure 1:**
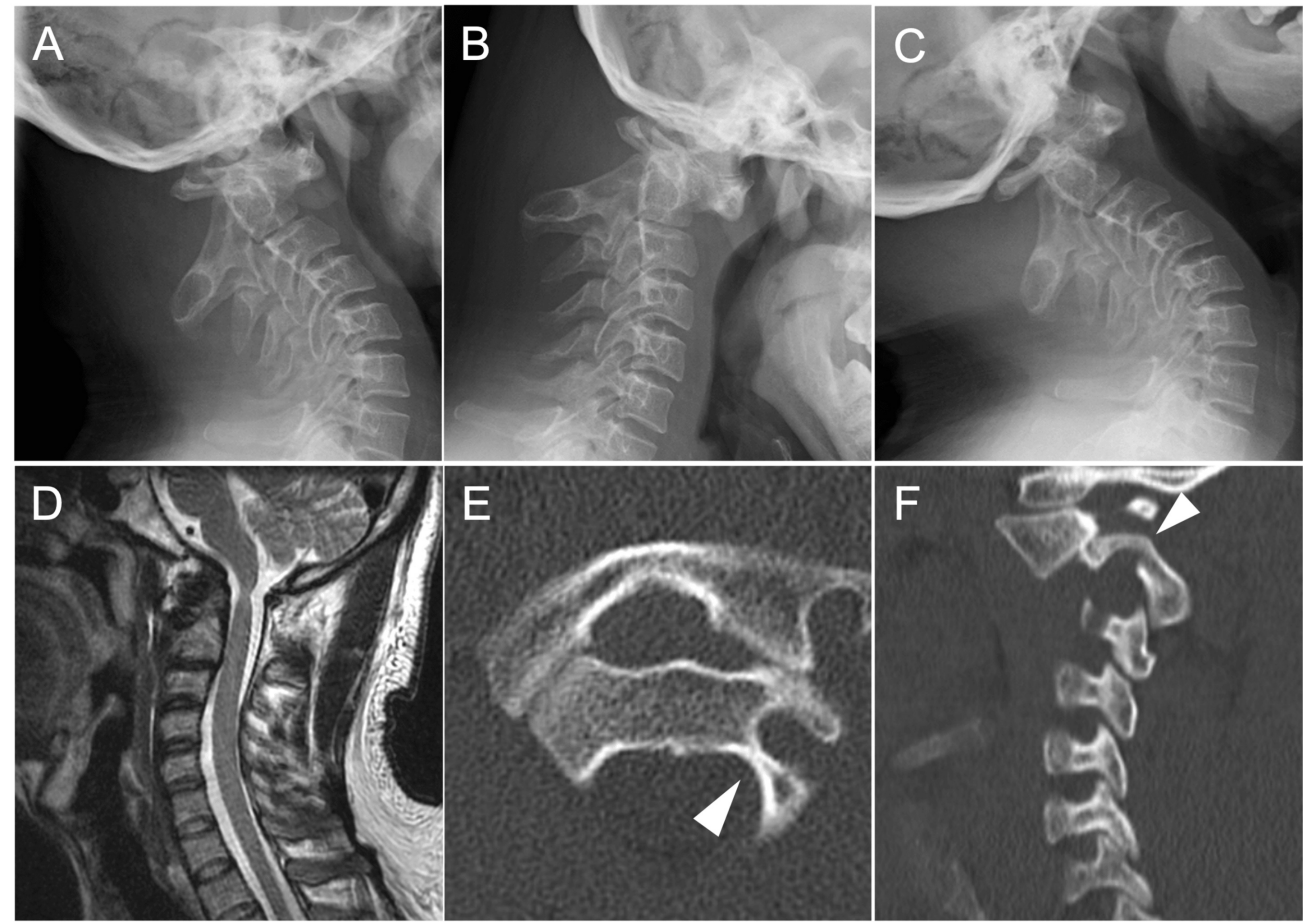
Preoperative imaging Lateral radiographs in neutral, flexion, and extension positions (A-C) demonstrate persistent atlantoaxial subluxation. T2-weighted sagittal magnetic resonance imaging (D) shows cervical cord atrophy with subtle intramedullary hyperintensity at C1-2. Contrast-enhanced computed tomography and computed tomography angiography (E, axial; F, sagittal) delineate a left high-riding vertebral artery and a markedly narrowed left C2 pedicle osseous corridor (white arrowhead) that precluded a left C2 pedicle screw trajectory.

At presentation, lateral radiographs in neutral, flexion, and extension demonstrated persistent anterior AAS without reduction on extension. T2-weighted sagittal MRI showed cord atrophy with subtle intramedullary hyperintensity at the C1 level. CT angiography revealed a left HRVA (C2 isthmus height: 2.2 mm, C2 internal height: 3.1 mm) and a narrowed C2 bony corridor (Figure 1). According to the Klepinowski classification of HRVA, this anatomy corresponds to a type 1 [10]. Taken together, these findings indicated that AAS is complicated by an HRVA with cervical myelopathy.

**Figure 2:**
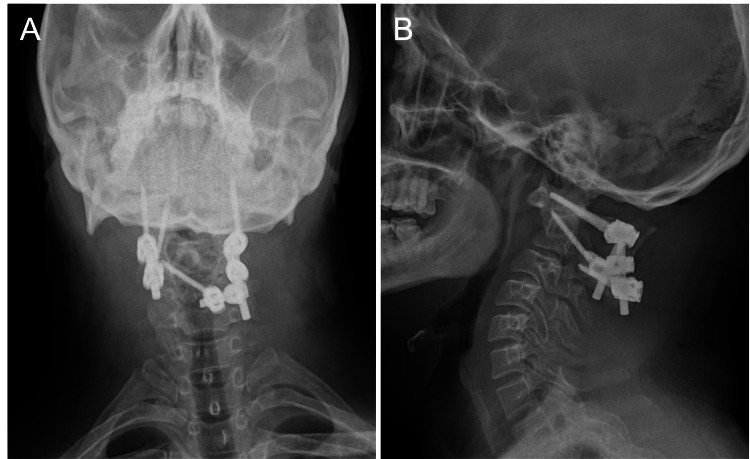
Postoperative radiographs Postoperative anteroposterior (A) and lateral (B) cervical spine radiographs demonstrate the final posterior C1-2 fixation construct, including C1 lateral mass screws and a hybrid C2 fixation (ipsilateral C2 pars and intralaminar screws with a contralateral C2 pedicle screw) connected by rods. The images confirm good C1-2 alignment after reduction and instrumentation.

**Figure 3:**
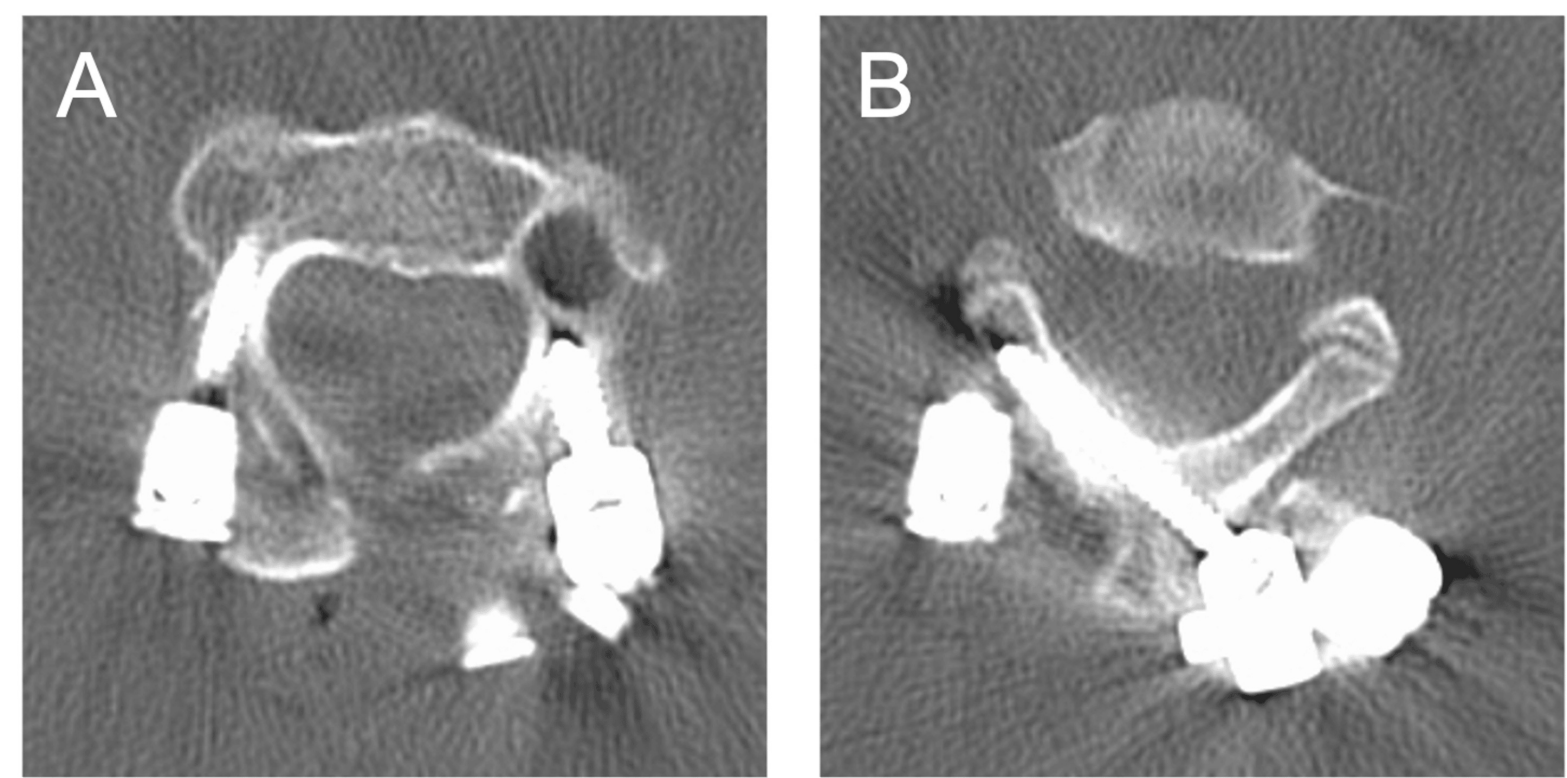
Postoperative CT Axial CT images at slightly different C2 levels (A, B) demonstrate satisfactory screw trajectories: panel A (more cranial) shows the large-diameter left C2 pars screw together with the contralateral C2 pedicle screw, whereas panel B (more caudal) shows the left C2 intralaminar screw.

He subsequently underwent posterior C1-2 fusion. Given the narrowed C2 pedicle corridor on the left, we selected a hybrid construct consisting of a cranial C2 pars screw (diameter: 4.5 mm, length: 10 mm) oriented for medial cortical purchase and a caudal C2 ILS (diameter: 3.5 mm, length: 28 mm) on the left, combined with a contralateral C2 pedicle screw (diameter: 3.5 mm, length: 28 mm) and bilateral C1 lateral mass screws (diameter: 3.5 mm; lengths: 26 mm and 28 mm). Reduction of the anterior displacement was achieved by applying a controlled C2 reduction force during instrumentation, after which the C1-2 laminae were decorticated and grafted with iliac crest autograft. The procedure was performed using a conventional freehand technique; intraoperative C-arm fluoroscopy was used to confirm implant position without the use of advanced navigation systems. Immediate postoperative anteroposterior and lateral radiographs confirmed the final construct and good C1-2 alignment (Figure 2). Early postoperative CT confirmed the planned screw trajectories (Figure 3).

After surgery, neck pain, dexterity, and gait gradually improved, and he returned to independent activities of daily living. As with the preoperative evaluation, formal objective scoring could not be performed due to his cognitive impairment. At approximately 12 months after surgery, he remained independent with no implant-related complications, and solid bony fusion was achieved without any signs of instability or implant loosening.

## Discussion

We report HRVA-complicated AAS managed with a large-diameter C2 pars screw combined with a C2 ILS on the HRVA side. This case illustrates that a pars-ILS hybrid construct can provide a safe anchor configuration when a C2 pedicle trajectory is judged hazardous, with no apparent vertebral artery or implant-related complications and with satisfactory early clinical recovery. In HRVA, the bony corridor for C2 pedicle or transarticular screws is reduced, which narrows the safety margin around the vertebral artery and requires a trade-off between vascular safety and construct rigidity [1-3]. Pars screws shorten the screw path and may reduce vascular risk, but can be mechanically inferior to pedicle screws if cortical purchase and diameter are not carefully optimized [8]. Constructs that include C2 laminar elements can achieve stiffness that approaches pedicle-based constructs in some loading planes, although pedicle screws generally retain higher pullout strength [4,5,7].

In our patient, combining a large-diameter pars screw with an ILS allowed load sharing across multiple cortices and made use of two distinct cortical trajectories when a pedicle screw was unsafe. Screw diameter and cortical purchase are key determinants of pullout strength and construct robustness in cervical fixation models [6,7]. A larger pars screw can increase thread engagement at dense cortical bone along the medial pars, while the ILS adds a transverse cortical path that resists flexion-extension and axial rotation. This strategy of exploiting dense cortical beds is conceptually similar to paravertebral foramen screw constructs that seek secure cortical purchase when pedicle corridors are hazardous [11,12]. In this case, improvement in canal reserve after reduction and fixation, together with neurological and functional recovery, suggests that this hybrid configuration provided adequate stability within a safety-oriented framework.

Several alternative C2 anchors are available for HRVA, including standalone pars screws, ILSs, subfacetal screws, and medial in-out-in screws [13-16]. Clinical and morphometric studies indicate that pars and ILSs can be placed with low vertebral artery risk in HRVA but are each constrained by local anatomy, and both tend to provide lower stiffness than conventional pedicle screws when used alone [4,5,7,8,13]. Subfacetal screws can obtain relatively long purchase above the vertebral artery in selected patients with sufficient internal height beneath the C2 superior articular facet, but this technique is applicable only when specific facet morphology is present and places the entry point close to the vertebral artery [16]. Medial in-out-in techniques and medial window screws can achieve strong multicolumn fixation and favorable biomechanical performance but intentionally breach the medial cortex toward the spinal canal and are technically demanding, usually requiring advanced imaging and considerable experience [14,15]. In a setting without routine navigation and in a patient with HRVA and limited C2 bony corridors, we considered that a technically simpler hybrid construct that combines a large-diameter pars screw with an ILS offered a practical compromise, prioritizing vertebral artery safety while still achieving good early stability and clinical improvement. However, this technique has some downsides. Biomechanically, the combination of pars and ILSs is generally inferior to bilateral C2 pedicle screws in terms of overall construct rigidity and pullout strength. Therefore, this hybrid construct should be considered an alternative or salvage option rather than a routine replacement when safe C2 pedicle trajectories are available. Furthermore, regarding rod connection, the entry points for the pars and ILSs differ significantly in both the coronal and sagittal planes. To facilitate rod assembly, the use of polyaxial screw heads and meticulous three-dimensional rod contouring is essential.

## Conclusions

In patients with HRVA who require C2 fixation, a large-diameter C2 pars screw combined with a C2 ILS can provide a safe C2 anchor configuration and good early clinical stability. This hybrid construct may serve as a practical, safety-oriented alternative for selected HRVA cases in which conventional C2 pedicle screws are not feasible.
